# Mercury in the Body of the Most Commonly Occurring European Game Duck, the Mallard (*Anas platyrhynchos* L. 1758), From Northwestern Poland

**DOI:** 10.1007/s00244-012-9860-6

**Published:** 2013-01-24

**Authors:** Elzbieta Kalisinska, Danuta I. Kosik-Bogacka, Piotr Lisowski, Natalia Lanocha, Andrzej Jackowski

**Affiliations:** 1 Department of Biology and Medical Parasitology, Pomeranian Medical University, Powstancow Wielkopolskich 72, 70-111 Szczecin, Poland; 2 Section of Toxicology and Bioanalytics, Department of Civil and Environmental Engineering, Koszalin University of Technology, Sniadeckich St. 2, 75-453 Koszalin, Poland; 3 Department of Zoology and Beekeeping, West Pomeranian University of Technology, Doktora Judyma St. 20, 71-466 Szczecin, Poland

## Abstract

The aim of this study was to determine the concentration of mercury (Hg) in liver (L), kidney (K), breast muscle [BM (*musculus pectoralis major*)], breast feathers (BF), and stomach contents (SC) of mallard (*Anas platyrhynchos* L. 1758). Among the edible parts of mallard, the greatest concentrations of Hg were observed in K and L, although they did not exceed 1.5 mg/kg dry weight (dw). Average concentrations in K, L, and BM were 0.27, 0.25, and 0.13 mg/kg dw, respectively. Significant correlations were observed between Hg concentrations in BM and K and in BM and L (*r*_s_ = 0.92) as well as between Hg concentrations in these tissues and BF. In addition, we found significant correlations between Hg concentrations in SC and BM (*r*_s_ = 0.72) and in L and K (*r*_s_ = 0.55). In conclusion, mallard exhibits a measurable response to environmental Hg pollution and meets the requirements of a bioindicator.

Mercury (Hg) is one of the most toxic trace metals affecting living organisms. In nature, this element is present in the greatest concentrations in bituminous shales, basic crystalline rocks, clay and peat soils, oil, and coal. Hg enters the environment as a result of volcanic eruptions and also as a result of human activity, the latter being mainly associated with municipal waste, cement, paints, dental amalgams, the steel industry, and the burning of fossil fuels. Global Hg emissions in 2005 were 1930 tonnes, of which 1480 tonnes were from anthropogenic sources (Arctic Monitoring and Assessment Programme/United Nations Environment Programme 2008). In Europe, Poland ranks second to Russia in emitting Hg into the environment, nearly 16 tonnes/y, which represents approximately 10 % of total European emissions (Arctic Monitoring and Assessment Programme/United Nations Environment Programme 2008; Debski et al. 2009). In Poland during the period 1990 to 2007, after socioeconomic transformation, Hg emissions decreased by more than half (Debski et al. 2009).

Mercury and its compounds enter the aquatic environment by way of wastewater, groundwater, and surface water runoff as well as directly from the air through precipitation and dry deposition. Mercury is extensively bound to sediments and undergoes methylation in conjunction with the activity of microorganisms. All forms of Hg, whether elemental, inorganic, or organic, and in particular its most widespread form, methylmercury (MeHg), are highly toxic to aquatic organisms, although there is some variation in sensitivity between different species (Wiener et al. 2003). As a result of biomagnification along the food chain, MeHg reaches its highest concentrations in organisms that are at the top of the trophic pyramid, i.e., predators (United States Environmental Protection Agency 1997; Wiener et al. 2003).

Terrestrial vertebrates absorb Hg through the skin and their respiratory and digestive systems (Graeme and Pollack 1998). In the digestive tract, 95 % of MeHg is absorbed, whereas metallic Hg and its inorganic compounds are poorly absorbed. Mercury accumulates in internal organs, including the brain (Castoldi et al. 2001; Davis et al. 1994; Graeme and Pollack 1998). Marine mammals and birds accumulate large amounts of Hg in L, where MeHg undergoes demethylation (Ikemoto et al. 2004).

Significant amounts of Hg are also observed in kidneys (K). Mercury in vertebrates is removed through the digestive and excretory systems. After biotransformation in the liver (L), Hg-containing metabolites migrate in the bile and are excreted by way of the feces (Boening 2000). In birds, significant amounts of Hg are removed during molting (Burger and Gochfeld 1997). It has been estimated that bird plumage contains from 55 to >90 % of total Hg (THg), mainly MeHg, accumulated in the body (Agusa et al. 2005; Spalding et al. 2000). In birds, the main effects of MeHg poisoning are manifested by decreased reproductive success associated with increased mortality of embryos and a greater number of unfertilized eggs (Wolfe et al. 1998). The neurotoxic effects of Hg on birds are manifested by motor ataxia and behavioral disorders (Heinz 1979). Moreover, at the biochemical level, the toxicity of Hg is exhibited by an adverse effect on the immune system, thus contributing to the induction of autoimmune aggression (*e.g.*, leading to K damage), and MeHg induces the production of free radicals and their associated oxidative stress (Griem and Gleichmann 1995; Hultman and Hansson-Georgiadis 1999; Ji et al. 2006). In general, the highest Hg concentrations have been reported in L and K of piscivorous birds, although in highly polluted regions equally high concentrations are found in omnivores (Thompson and Furness 1989).

The mallard (*Anas platyrhynchos* L. 1758) is the most common breeding duck and the most commonly hunted duck in Europe, generally, as well as in Poland (Cramp and Simmons 1983). Poland is the country with the third highest number of mallards in Europe after Germany and the Netherlands. The size of the national breeding population of the species has been estimated to be 200,000 to 400,000 pairs, and the status of the mallard as a breeding bird is stable and shows no major fluctuations (BirdLife 2004). Mallards inhabit almost all types of wetland area as well as both freshwater and saltwater inland reservoirs. As an omnivorous species, they feed on both land and water plants, aquatic invertebrates, and small aquatic vertebrates [amphibians (and their tadpoles) and fish]. Mallards belong to the group of dabbling ducks, and they collect a large proportion of their food from the surface of the water or the bottom of shallow reservoirs (Hoyo et al. 1992).

As a member state of the European Union (EU), Poland is obliged to monitor chemical (and other) residues in animals and animal products, including game animals and their products. However, the Ministry of Agriculture and Rural Development (Official Journal 2006, No 147, pos. 1067), in referring to European Council Directive 96/23 EC, mentions only three species of animal: the wild boar (*Sus scrofa* Linnaeus 1758), the red deer (*Cervus elaphus* Linnaeus 1758), and the roe deer (*Capreolus capreolus* Linnaeus 1758). According to these documents, two groups of substances are measured in game animals: pesticides and polychlorinated biphenyls (group B3) and toxic elements, including Hg (group B3c). Within a few years of starting to monitor these substances, in game mammals (which are typical terrestrial organisms), excess levels of Hg were recorded only in a few individual L samples (Swiader 2007; Zmudzki et al. 2005). The aim of this study was to investigate Hg concentrations in liver (L), kidney (K,) breast muscle [BM (*musculus pectoralis major*)], breast feathers (BF), and stomach contents (SC) of mallards.

## Materials and Methods

### The Research Area

**Fig. 1:**
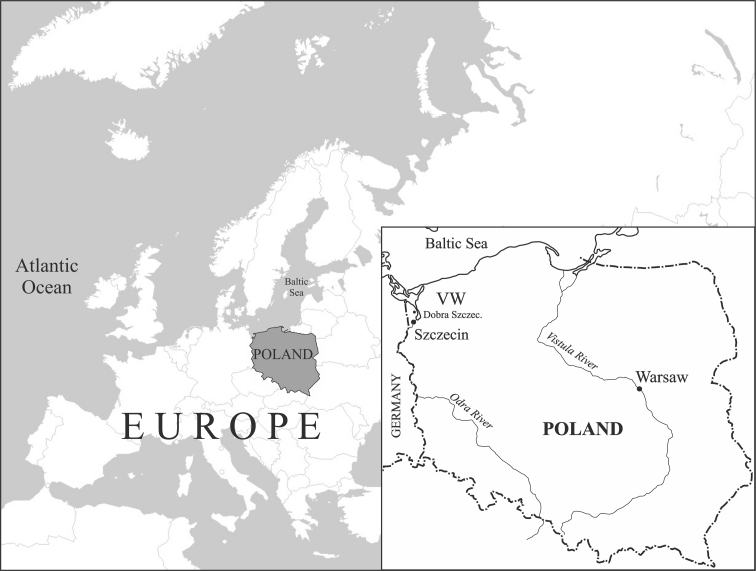
Location of study areas in northwestern Poland

The research material was collected in autumn (September to November) of 2005 and 2006 from the province of West Pomerania in northwestern Poland, whose capital, Szczecin (53° 25 ‘57’’ N, 14° 33’19’’ E), is inhabited by approximately 400,000 people. The hunting grounds from which the ducks were taken include five field ponds located approximately 10 to 20 km from the centre of Szczecin in the municipality of Dobra Szczecinska, northwest of Szczecin (Fig. 1). Industrial facilities, which are significant emitters of Hg, are located in the capital and its immediate vicinity. The most significant of these include power and heating plants, which use coal as a raw material; chemical plants; and ship repair yards.

It was estimated that in 2007, the atmospheric Hg deposition in West Pomerania was 515 kg (Debski et al. 2009). A large part of the area lying to the northwest of Szczecin is subject to legal protection under the Protection of Nature Act 2004 (Journal of Laws 2004, No 92, pos. 880) under the provisions relating to Special Protected Areas (SPAs): Wkrzanska Refuge (PLB320014) and Swidwie Lake (PLB320006) (Journal of Laws 2008, No 198, pos. 1226) are part of the European Union’s ecological network (Natura 2000). These SPAs partially overlap with the area of research.

In Poland, Hg has been determined only in water samples and sediments coming from large rivers and lakes covered by National Environmental Monitoring since the 1990s. Because the mallards examined in this study were associated with small water bodies, we decided to analyse these water and sediment samples to provide at least a basic picture of Hg contamination in these areas. To determine the concentration of Hg in sediment and water samples from the five field ponds where the ducks lived, we collected a total of ten surface sediment samples and five water samples. Surface sediment samples (0 to 10 cm) were collected with large-mouth polyethylene bottles. After separating the organic material from the gravel and sand that was present, the samples were dried to a constant weight at 50 °C and then homogenized in a planetary mill for determination of THg concentrations. In these sediment samples, Hg concentrations ranged from 0.01 to 0.20 mg/kg dw [arithmetic mean (AM) ± SD were 0.11 ± 0.06 mg/kg dw], and the sediment samples were classified as lightly polluted [class I (Bojakowska and Sokolowska 1998)].

The concentration of Hg in the water taken from the same ponds was at the threshold of detection (<0.01 ng/l). In the EU, the maximum permissible mean Hg concentration in inland surface waters in 0.07 μg/l (Journal of Laws UE 2008, L 348/84); thus it may be assumed that Hg concentrations were negligible in the analyzed field ponds.

### Sampling

This study used 50 hunted mallards (*A. platyrhynchos*), which were subject to morphometric examination at a laboratory. We determined their sex and age, body weight and body length (from the base of the beak to the tail), stroke length, and folded left wing length in accordance with Dzubin and Cooch (1992). On the basis of the degree of development of the ducks’ bursa of Fabricius, they were classified into two age categories: the first year of life (*i.e.*, immature [IM]) and adult (AD) (Hochbaum 1942; Hohman and Cypher 1986; Siegel-Causey 1990). The birds studied included 41 IM [26 female (F) and 15 male (M)] birds and 9 AD (3 F and 6 M) birds. Procedures of sample preparation for analyses were different depending on the respective type of sample.

#### BM and Organs

From each duck we collected L, K, and BM. These samples (approximately 10 g) were dried to a constant weight at 50 °C for approximately 50 or 60 days and were weighed (to 0.1 mg; Sartorius BP221S balance) three times to constant weight, which made it possible to determine weight-based sample water content. Subsequently, the samples were pulverised in a Planetary Mono Mill Pulverisette 6 (Fritsch GmbH, Germany). This procedure is consistent with that used by other researchers (Houserova et al. 2005; Kalisinska et al. 2009, 2010).

#### Breast Feathers

We collected BF samples from each duck. The BF were prepared for analysis according to Burger and Gochfeld (1993). To remove surface contamination, the BF samples were rinsed in acetone, centrifuged, and then washed in distilled water. The dried BF were then cut into small pieces (approximately 1 to 3 mm^2^).

#### Stomach Contents

We collected SC samples from 25 of the ducks. After separating the organic material from the gravel and sand that was also present, the samples were dried to a constant weight at 50 °C and then homogenized in a planetary mill.

### Determination of THg Concentrations

THg concentration in all examined samples (L, K, BM, BF, and SC) was determined using atomic absorption spectroscopy (Lucia et al. 2010) at the Department of Environmental Management and Protection at the West Pomeranian University of Technology in Szczecin. The assays were run in an AMA 254 Hg analyser (Altach Ltd, Czech Republic). The AMA 254 detection limit and linear range were 0.01 and 0.05 to 40 ng, respectively. This method requires no acid digestion. Portions of the dried samples weighing ≤0.1 g were placed directly in the nickel boat. The portions were dried for 100 seconds at 200 °C and then decomposed for 320 seconds at 550 °C; the waiting time for analysis was 45 seconds. Mercury was retained in a gold trap and released by the heating method. Mercury concentrations were calculated with reference to both the dry weight (dw) and wet weight (ww) of the material being analysed.

The precision of the analytical procedure was maintained by determination of the Hg concentration in two certified biological materials: lyophilized bovine L (BCR 185) and lyophilized porcine K (BCR 186) [Commission of European Communities, Community Bureau of Reference (Table 1)]. The percentage recoveries were 102 and 96 %, respectively.

**Table 1:** Concentrations of Hg (mg/kg dw) in certified reference materials

	Bovine L BCR 185 (*n* = 6)		OD/RV (%)	K BCR 186 (*n* = 7)		OD/RV (%)
	RV	OD		RV	OD	
Hg	0.044 ± 0.003	0.045 ± 0.005	102	1.97 ± 0.04	1.90 ± 0.06	96

*RV* reference value, *OD* investigators determination

### Statistical Analysis

We calculated water content in L, K, and BM of mallards as a percentage and established the statistical characteristics of biometric measurements and chemical analyses. We determined the AM and SD in relation to the size of the ducks and their organs. To investigate possible relationships between duck body mass and the mass of L and K and between the size of L and K, we calculated the Pearson correlation coefficients. In addition, we determined the appropriate regression equations for these relationships, which showed that they had strong relationships (*r* > 0.70). We also determined the median and AM and SD (AM ± SD) for concentrations of Hg. To determine whether the expected distribution of our results corresponded with the normal distribution, we used Shapiro–Wilk test (*p* < 0.05) for both the raw data and the data that was subjected to logarithmic transformation (log_10_).

To analyse the differences between the selected biometric parameters and the values of Hg concentrations in the samples obtained from the mallards (including analysing the differences taking into account the categorisation of birds by sex and age), we used Student *t* and nonparametric Mann-Whitney tests (*p* < 0.05). The strength of the relationships between the Hg concentrations in BF, and in SC was estimated using the Spearman correlation coefficient (*r*_s_, *p* < 0.05 and *p* < 0.001). Statistical analyses were performed using the statistical package Statistica 8.0 (StatSoft, Poland).

## Results

### Mallard Morphometry

The average mallard weight was 1,020 g. Data on the body dimensions and organs of mallards included in Table 2. On average, the F birds weighed approximately 130 g less than M birds and the IM birds approximately 200 g less than AD birds (Table 2). The kidney mass of the mallards examined was between 10 and 11 g, which was typically 3 times less than the mass of L, which on average was approximately 32 g but was sometimes as high as >66 g. There was a significant correlation between mallard total body weight and L weight (*r* = 0.55, *p* <0.05). K and L accounted for approximately 1 and 3.1 %, respectively, of body weight.

**Table 2:** Body dimensions and organ relative weights of mallards from northwestern Poland

Age or sex group and no. of individuals in each group (n)		Body dimensions				Organ dimensions	
		Weight (g)	Length (cm)	Leaf length (cm)	*Tarsometatarsus* length (mm)	K (g)	L (g)
IM (*n* = 41)	AM ± SD	985 ± 122	49 ± 3	27 ± 1	43 ± 2	10 ± 3	31 ± 8
	Minimum–maximum	645–1170	45–55	25–29	39–49	5–16	15–47
AD (*n* = 9)	AM ± SD	1187 ± 162	51 ± 4	29 ± 2	46 ± 2	11 ± 2	36 ± 13
	Minimum–maximum	870–1395	45–57	26–31	43–48	7–13	22–66
M (*n* = 21)	AM ± SD	1098 ± 165	52 ± 2	28 ± 1	45 ± 2	11 ± 3	35 ± 12
	Minimum–maximum	645–1395	47–57	27–31	40–49	7–16	15–66
F (*n* = 29)	AM ± SD	966 ± 111	48 ± 2	27 ± 1	43 ± 2	10 ± 3	30 ± 5
	Minimum–maximum	760 –1205	45–54	25–29	39–46	5–15	17–43
Total (*n* = 50)	AM ± SD	1021 ± 150	50 ± 3	28 ± 1	44 ± 2	10 ± 3	32 ± 9
	Minimum–maximum	645–1395	45–57	25–31	39–49	5–2	15–66

### Hg Concentration in Biological Samples Taken from the Mallards

The average percentages of water content in L, K, and BM were approximately 72, 78, and 77 %, respectively. Basic data on the Hg concentrations measured is listed in Table 3. The distribution of Hg concentrations obtained did not show the usual characteristics of distribution (*p* < 0.05 for the Shapiro-Wilk test), but after logarithmic transformation the data assumed distribution characteristics that were consistent with normal distribution.

**Table 3:** Total Hg concentration in BM, L, K, and BF (mg/kg dw) in mallards from northwestern Poland

Age and sex group and no. of individuals in each group (*n*)		BM	L	K	BF
IM (*n* = 41)	Median	0.114	0.203	0.270	0.429
	Q_L_–Q_U_	0.034–0.190	0.082–0.345	0.105–0.385	0.140–0.833
	AM ± SD	0.146 ± 0.175	0.257 ± 0.225	0.286 ± 0.281	0.649 ± 0.724
	CV	119.7	87.4	99.3	111.3
	Minimum–maximum	0.011–0.938	0.019–0.966	0.029–1.499	0.037–3.475
AD (*n* = 9)	Median	0.062	0.189	0.190	0.360
	Q_L_–Q_U_	0.030–0.101	0.058–0.326	0.096–0.254	0.195–0.857
	AM ± SD	0.074 ± 0.057	0.205 ± 0.154	0.199 ± 0.138	0.561 ± 0.542
	CV	77.5	74.9	69.7	96.6
	Minimum–maximum	0.008–0.201	0.016–0.468	0.010–0.4721	0.071–1.719
M (*n* = 21)	Median	0.115	0.248	0.270	0.513
	Q_L_–Q_U_	0.034–0.190	0.118–0.361	0.190–0.331	0.342–0.837
	AM ± SD	0.152 ± 0.197	0.273 ± 0.216	0.315 ± 0.306	0.620 ± 0.508
	CV	129.3	79.3	98.8	81.6
	Minimum–maximum	0.012–0.938	0.019–0.958	0.030–1.499	0.042–1.972
F (*n* = 29)	Median	0.071	0.166	0.146	0.357
	Q_L_–Q_U_	0.027–0.146	0.068–0.343	0.076–0.351	0.125–0.833
	AM ± SD	0.120 ± 0.134	0.230 ± 0.213	0.237 ± 0.224	0.643 ± 0.807
	CV	112.2	92.7	94.6	125.4
	Minimum–maximum	0.008–0.609	0.016–0.966	0.010–1.107	0.037–3.475
Total (*n* = 50)	Median	0.096	0.198	0.248	0.393
	Q_L_–Q_U_	0.030–0.178	0.074–0.345	0.096–0.351	0.140–0.837
	AM ± SD	0.133 ± 0.162	0.248 ± 0.213	0.270 ± 0.262	0.634 ± 0.691
	CV	121.8	86.0	97.9	108.9
	Minimum–maximum	0.008–0.938	0.016–0.966	0.010–1.499	0.037–3.475

*Q*_*L*_ lower quartile, *Q*_*U*_ upper quartile, *CV* coefficient of variation (%)

Mercury concentrations were greater in samples from IM birds than those from AD birds, but these differences were not confirmed statistically. In addition, no statistically significant differences were observed between Hg concentrations in samples taken from M compared with F birds (Mann-Whitney* U* test, *p* > 0.05). Therefore, to determine the comparison as well as the relationship between the Hg concentrations of a variety of mallard biological material, data were collected from all of subjects in the study (*n* = 50).

The lowest concentration of Hg was found in BM and differed significantly from Hg concentrations in L, K, and BM (*p* < 0.01). In BF, the Hg concentration was much greater than that in either L or K (*p* < 0.01). We found no statistically confirmed differences between L and K Hg concentrations in the mallards (*p* > 0.05). The average Hg concentrations (medians) in the material analysed were as follows: BF > K = L > BM.

The highest correlation coefficients were found between Hg concentrations in BM and L and those between BM and K (in both cases *r*_s_ = 0.92, *p* < 0.001) as well as those between L and K (*r* = 0.90, *p* < 0.001). There was also a slightly less pronounced correlation between Hg concentrations in L and K, those in K and BF (in both cases *r*_s_ = 0.80, *p* < 0.01), and those in BM and BF (*r* = 0.77, *p* < 0.001). In the edible parts of the mallard, the breast muscles (BM) and the L, the average Hg concentrations were 0.021and 0.053 mg/kg ww, respectively.

### Hg in SC of Mallards

In SC, the average Hg concentration was low and amounted to only 0.01 mg/kg dw, with individual samples ranging from below detection limit to 0.04 mg/kg. The median and AM Hg concentrations did not differ from each other, and in both cases their value was 0.01 mg/kg dw (at SD 0.01 mg/kg). The consistency of the distribution of results in our study with the expected normal distribution was shown by Shapiro-Wilk test (*p* <0.05). We found a correlation between Hg concentrations in SC and BM (*r* = 0.72, *p* <0.001), L, and K [*r* approximately 0.55 (*p* < 0.05)] but not between those in SC and BF.

## Discussion

The mallard is an omnivorous species consuming both animal and plant material coming from the water and land. This duck belongs to the water trophic net and takes part in Hg transport, including that of MeHg, from water to terrestrial ecosystems. Depending on the degree of Hg pollution of the local environment and the Hg content in food, the mallard absorbs varied amount of this toxic metal similar to other anseriform species (Evers et al. 2005; Hall et al. 2009; Kalisinska et al. 2012). In the postbreeding season, researchers sometimes observe relationships between age classes (but usually not between sex groups) and Hg concentrations in L and other tissues. There is a tendency for greater Hg accumulation in AD than IM birds (Burger 2007; Conover and Vest 2009; Custer and Custer 2000; Evers et al. 2005; Lindsay and Dimmick 1983; Parslow et al. 1982). In our study in northwestern Poland, there was no significant correlation between Hg concentrations in the various organs and the age and sex of the mallards. This result is similar to those obtained with various *Anseriformes* from Europe and North America, including the mallard (Florijancic et al. 2009; Kalisinska et al. 2010; Vermeer et al. 1973).

Three internal tissues (L, K, and BM) and one external tissue (BF) are commonly used to determine Hg exposure to birds (Eisler 1987; Evers et al. 2005; Ikemoto et al. 2004; Parslow et al. 1982; Wiener et al. 2003). In all of the examined biological materials of mallards from northwestern Poland, we detected low Hg concentrations. The average Hg concentrations in mallard L, K, BM, and BF were 0.19, 0.24, 0.010, and 0.393 mg/kg dw, respectively. As the most important detoxifying organs, L and K (to a lesser extent) are the most commonly analyzed internal tissues to determine Hg concentration in birds, including the mallard (Aazami et al. 2012; Stickel et al. 1977; Burger and Gochfeld 1985; Eisler 1987; Evers et al. 2005; Kalisinska et al. 2012; Vest et al. 2009; Wiener et al. 2003). The “7:3:1 rule” is often used as a reference ratio for Hg levels in avian L (ww): BF (fresh weight): BM (ww), especially in moderately to severely Hg-exposed birds (Appelquist et al. 1984; Evers et al. 2005). In the case of the mallards in this study, the ratio of Hg concentrations in dry-weight tissues was 2:4:1 and thus differed from the above-mentioned wet-weight ratio, probably due to the fact that the examined duck population living near Szczecin was not exposed to environmental Hg.

Considerably greater hepatic Hg concentrations than those found in our study were reported by Parslow et al. (1982) in mallards from the Ouse Washes in England (Table 4). Lucia et al. (2010), measuring Hg concentrations in L and K from one mallard from the French Atlantic coast, found approximately 15 mg/kg and approximately 40 mg/kg dw Hg, respectively (approximately 3.7 mg/kg and approximately 8 mg/kg ww, respectively), which probably was due to the mallard consuming a diet rich in fish. It should also be noted that in >80 % of cases as methylmercury (MeHg), which is almost completely absorbed by the gastrointestinal tract of birds (Karasov 2011). In addition, MeHg constitutes a large proportion of THg in BM of birds. The proportion of MeHg to THg has been estimated to be 70 to 90 % depending on the species (Scheuhammer et al. 2007). Mercury concentrations in muscle are generally examined in waterfowl because MeHg concentrations in this tissue, the largest edible part of the duck, pose a risk to human health (Aazami et al. 2012; Cohen et al. 2000; Gerstenberger 2004; Hall et al. 2009; Kalisinska et al. 2010). Mean Hg concentrations in muscle of wild mallard from various parts of the world have ranged from 0.008 to 25.52 mg/kg dw (Table 4). As shown by Binkowski et al. (2012), muscle Hg concentrations are very similar regardless of the type of muscle *e.g.* in BM, Hg ranges from 0.021 to 0.025 mg/kg ww, and in leg muscle it ranges from 0.020 to 0.024 mg/kg ww. Our data set supports other findings that BM Hg levels are generally less than those of L and K. In Europe, twice greater Hg levels in muscle of mallards than those found in our study were recorded in Finland, Gulf of Bothnia (Koivusaari et al. 1976). A similar value was found in one duck from the French Atlantic coast (Lucia et al. 2010), but the value was 3 to 4 times lower in mallards from southern Poland and Slovakia (Binkowski et al. 2012; Gasparik et al. 2010) (Table 4). Mercury concentrations in L and muscle of mallards several times greater than those found in mallards of northwestern Poland have been observed in mallards living in North America (Table 4). Correspondingly, results obtained in mallards from an Hg-polluted region of Canada in the 1970s showed a muscle Hg concentration of almost 25 mg/kg dw (Vermeer et al. 1973). The cause of such large accumulations of Hg in these mallards was probably the widespread use of Hg-containing pesticides, from which Hg was then absorbed by mallards by way of their food. Since the 1990s, the muscles of mallards surveyed in different parts of the world, including Canadian prairie ponds (Hall et al. 2009), have contained low levels of Hg (<0.10 mg/kg ww), with the exception of one population living in the highly Hg-polluted Salt Lake (Utah, USA), in which muscle Hg levels were >0.28 mg/kg ww (Table 4).

**Table 4:** Comparison of total mercury (THg) concentrations (mg/kg dw and ww) in wild and domestic ducks (*A. plathyrynchos*) from European countries and other parts of the world

Species	n	Mean	dw or ww	BM	L	K	Study area	Reference
*A. platyrhynchos* from European countries								
50		M	dw	0.096	0.198	0.248	Poland, NW	This study
				0.030–0.178	0.074–0.345	0.096–0.351		
			(ww)	(0.024)	(0.059)	(0.050)		
10 IM		GM	dw	0.015	0.033	0.045	Poland, NW	Lisowski and Kalisinska (2006)
				0.011–0.190	0.019–0.328	0.029–0.401		
			(ww)	(0.004)	(0.010)	(0.009)		
41 IM		GM	dw	0.114			Poland, Western Pomerania	Kalisinska et al. (2010)
			(ww)	(0.028)				
5		M	ww	0.021			Southern Poland	Binkowski et al. (2012)
			(dw)	(0.005)				
52		AM	dw		1.2 ± 0.1		Great Britain, Ouse Washes	Parslow et al. (1982)
			(ww)		(0.36)			
5		AM	ww	0.05 ± 0.01			Finland, Gulf of Bothnia	Koivusaari et al. (1976)
			dw	(0.20)				
10		AM	ww		0.116 ± 0.069		Croatia	Florijancic et al. (2009)
			(dw)		(0.386 ± 0.230)			
68		AM	ww	0.009 ± 0.005			Slovakia	Gasparik et al. (2010)
			(dw)	(0.029 ± 0.018)				
1			dw	0.12	14.79	39.66	France, Atlantic coast	Lucia et al. (2010)
		AM	(ww)	(0.03)	(3.70)	(7.93)		
*A. platyrhynchos* from other parts of the world								
16		AM	ww	6.13 ± 1.28			Canada, NW Ontario	Vermeer et al. (1973)
				0.9–10.4				
			(dw)	(24.52 ± 5.12)				
32		Range	ww	0.002–0.434			Canada, Southern Saskatchewan	Hall et al. (2009)
			(dw)	(0.007–1.447)				
5M		GM	ww		0.06	0.07	USA, Wisconsin	Stickel et al. (1977)
			(dw)		(0.20)	(0.23)		
7			ww		0.324 ± 0.097		USA, New Jersey	Burger and Gochfeld (1985)
			(dw)		(1.080 ± 0.323)			
10		AM	ww	0.282			USA, Utah, Great Salt Lake	Scholl and Ball (2005)
				0.034–0.662				
			(dw)	(0.940)				
12 M		AM	ww	0.06 ± 0.06	0.47 ± 0.94		USA, Nevada	Gerstenberger (2004)
17 L				0.01–0.22	0.03–3.92			
			(dw)	(0.24 ± 0.24)	(1.57 ± 3.13)			
10		AM	ww	0.131 ± 0.161			USA, New York, Onondaga Lake	Meattey and Savoy (2012)
				<0.00–0.472				
			(dw)	(<0.00–1.884)				
18		AM	ww	0.01 ± 0.11	0.28 ± 0.03	0.26 ± 0.03	Iran, Caspian Sea wetlands	Aanzami et al. ( 2012)
			(dw)	(0.04 ± 0.44)	(0.93)	(1.04)		
*A. platyrhynchos* f. domestica (Pekin duck)								
5		AM	dw	0.00001	0.00013	0.00141	France	Lucia et al. (2008)
			ww	(0.000003)	(0.00004)	(0.0003)		
*A. platyrhynchos* f. domestica (Shaoxing duck)								
7		AM	ww	0.008 ± 0.003	0.022 ± 0.008		Shanghai-control area China	Ji et al. (2006)
			(dw)	(0.032)	(0.073)			
7		AM	ww	0.158 ± 0.034	4.465 ± 1.507		Guizhou province, China	
			(dw)	(0.527)	(17.86)			

*BM* breast muscle, *L* liver, *K* kidney, *AM* arithmetic mean, *GM* geometric mean, *SD* standard deviation, *SE* standard error, *M* median

The domesticated forms of mallard from Shanghai, China (an area of relatively lower Hg contamination)—Peking duck and Shaoxing duck—have Hg concentrations in L, K, and skeletal muscle that are several orders of magnitude lower than those recorded in wild mallards in various countries (Table 4). In contrast, in Shaoxing, free-range ducks living in the most contaminated province of China (Guizhou provence), Hg concentrations in L were highest compared with those in wild and domesticated breeds descended from *A. platyrhynchos* (Table 4), in which the L Hg concentration exceeded 17 mg/kg dw.

Until now, Hg concentrations in food products of animal origin in the EU (including Poland) is formally regulated only by laws relating to edible fish and shellfish (EU 2006 L 364). Maximum permissible Hg concentration (for human consumption) in the muscle of fish is 0.5 or 1.0 mg/kg ww, depending on the species. In none of the examined mallard from northwestern Poland did Hg L and BM concentrations exceed the 0.5 mg/kg ww threshold, although such cases have been noted in other parts of the world (Table 4).

Breast feathers are an important biological material in ecotoxicological studies. Between 70 and 90 % of Hg present in a avian body is deposited (as MeHg) in BF, with 30 to 50 % of the remaining Hg burden being located in the muscles, 8 to 19 % in L, and 2 to 6 % in K (Agusa et al. 2005; Evers et al. 2005). MeHg moves to BF as birds grow and always remains present there. For this reason, the general level of Hg in BF reflects the uptake of MeHg and the time of moulting (Evers et al. 2005).

The concentration of Hg in BF ranges from <1 to >1.5 mg/kg dw, although greater concentrations have been found in seabirds. It has been found in experiments that an Hg concentration in BF >5 mg/kg is connected to adverse effects in the body, which manifest as behavioural and reproductive disorders (Burger and Gochfeld 1997, 2004). In birds associated with terrestrial environments, Hg risk is indicated by an Hg concentration in BF >20 mg/kg, whereas concentrations <1 to 5 mg/kg are considered to result from the geochemical background (Scheuhammer 1991). It has also been found that the adverse effect of Hg on reproduction and behaviour of mallards is connected with a BF Hg concentration of 9 to 11 mg/kg (Eisler 1987). In the present study, none of the mallards examined had BF Hg concentration as high as 5 mg/kg, and only one had a level of approximately 3.5 mg/kg. Similar Hg concentrations in BF have been reported in other duck species that winter in northwestern Poland (*e.g.*, the goldeneye *Bucephala clangula*, greater scaup, and common pochard) (Lisowski and Kalisinska 2006).

The few available publications on Hg in ducks describe the relationship between Hg in various biological materials. Lisowski and Kalisinska (2005) found that in greater scaup, there is a strong correlation between Hg concentrations in muscles (M) and K, and also between K and BF (*r* = 0.68 and *r* = 0.91, respectively). In addition, in pochard they found a less pronounced but nevertheless significant correlation between Hg concentrations in BM and K and in the combinations BM–BF, L–K, and K–BF, with correlation coefficients ranging from 0.50 to 0.70. In piscivorous goosander from the same region of Poland, a correlation was observed between the Hg level in BM and K (*r* > 0.83) but not between L and K nor between L and BM (Kalisinska et al. 2010). In this study of mallards, we showed that there exist three strong correlations between Hg concentrations in various organs [BM and L, BM and K, and L and K (*r*_s_ approximately 0.90)] and three slightly weaker correlations [L and BF, K and BF, and BM and BF (*r*_s_ approximately 0.80)]. The large number of mallards (*n* = 50) analysed here, however, undoubtedly had a significant impact on the size of these correlation coefficients compared with data from greater scaup, pochard, and goosander (*n* < 20).

It has also been found that typical terrestrial environments and field ponds in northwestern Poland are only contaminated with Hg to a small extent but that the Szczecin Lagoon is an exception, with sediment containing between 4.5 and 14.5 times more Hg than sediment samples from field ponds in the same area (i.e., within approximately 20 to 25 km). The Hg concentration in the sediment of the lagoon ranges, on average, from 0.5 to 1.6 mg/kg dw depending on the exact location (Protasowicki and Niedzwiecki 2004).

In general, ecotoxicological studies focus on ducks found in large inland bodies of water and on the coast, where many species spend the winter. These environments have also been usually extensively researched in terms of their water and sediment quality. Much less frequently studied are small inland water reservoirs, which include the so-called ponds where ducks often stay, especially during the breeding season. Research into such small bodies of water has been performed in Canada concerning MeHg concentrations of water from lakes in the prairie pothole region of Saskatchewan and levels of Hg in the birds inhabiting such water bodies (Hall et al. 2009): This research found low levels of Hg in mallards, with the average THg concentration being approximately 0.06 mg/kg ww, which was similar to that found in birds from the clear water of the lakes, which ranged from 0.02 to >4 mg/l.

In biomonitoring studies, the aim of which is to assess indirectly the environmental level of heavy metals, the concentration of such metals in organs and BF of birds is often measured, but this is performed less frequently with regard to their skeletal muscles (Evers et al. 2005; Ji et al. 2006; Lucia et al. 2008; Wiener et al. 2003). Taking into account our results and those of other reports on mallard, a species with a large geographical range and with large numbers, it can be said that this species displays a measurable response to the amount of Hg in the environment. Moreover, existing studies in the field of experimental toxicology that deal with the effect of Hg on mallard biology enable proper interpretation of field study results. For these reasons, the mallard and its domesticated forms are a good bioindicator of inland environmental pollution of omnivorous organisms.
